# Late Onset* Streptococcus agalactiae* Meningitis following Early Onset Septicemia: A Preventable Disease?

**DOI:** 10.1155/2017/8418105

**Published:** 2017-10-01

**Authors:** Kam Lun Hon, King Hang Chan, Pak Long Ko, King Woon So, Alexander K. C. Leung

**Affiliations:** ^1^The Chinese University of Hong Kong, Prince of Wales Hospital, Shatin, Hong Kong; ^2^Faculty of Medicine, The Chinese University of Hong Kong, Shatin, Hong Kong; ^3^Department of Paediatrics, Prince of Wales Hospital, Shatin, Hong Kong; ^4^Department of Pediatrics, The University of Calgary, Calgary, AB, Canada

## Abstract

We report a neonate who presented with early onset* Streptococcus agalactiae* or group B streptococcus (GBS) septicemia within 24 hours of birth. After discharge at day 14, she went on to develop late onset GBS meningitis at 36 days of age. The infant was treated with intravenous antibiotics on both occasions and eventually discharged home with no apparent sequelae. We address issues associated with GBS infection in infancy including the demographics, risk factors, and the risk of late onset GBS meningitis following an early onset GBS infection. The major source of GBS in early onset GBS disease is maternal birth canal GBS colonization. On the other hand, nosocomial cross-infection is an important source of GBS in late onset disease. Penicillin remains the current treatment of choice for GBS infection. Given the rapid onset and progression within hours of birth and lack of an effective solution for preventing late onset GBS, administration of an effective GBS vaccine in pregnancy could provide a sensible and cost-effective solution in all settings.

## 1. Introduction

Neonatal infection with* Streptococcus agalactiae* or group B streptococcus (GBS) is serious, potentially preventable, and imminently treatable [1–7]. GBS is a common colonization agent in the maternal gastrointestinal and genital tract. The organism can spread from the mother to the neonate during vaginal delivery. The vast majority of neonatal infections caused by GBS occur within the first 6 days of life [1–3, 8, 9]. While early onset GBS infection (from birth to 6 days) often presents as sepsis, pneumonia, and, less commonly, meningitis, late onset GBS infection (from 7 days to 89 days) more commonly presents as bacteremia, meningitis, and, less commonly, other focal infections such as cellulitis, lymphadenitis, and bone/joint infection [1, 8, 10, 11]. Understanding the sources and mechanisms of infection is crucial in the prevention and treatment to reduce neonatal morbidity and mortality of this disease [1–3, 10–12]. This case illustrates that adequate antibiotic treatment for early onset GBS does not necessarily prevent late onset GBS disease. As GBS immunization is not currently available, known maternal GBS carriers with infants who had early onset GBS disease should be informed about risks of late onset GBS disease on discharge. These infants should be immediately assessed, should there be any suspicion of late onset GBS infection.

## 2. Case Report

A female neonate was born at 38 weeks of gestation to a 33-year-old primigravida mother following a spontaneous vaginal delivery. The mother had been well during pregnancy and antenatal GBS screen was negative. There was prolonged rupture of membrane of over 32 hours. Apgar scores were 9 and 9 at 1 minute and 5 minutes, respectively. Her birth weight was 3.035 kg (25th percentile), length 49 cm (25th percentile), and head circumference 32.5 cm (10th percentile). Shortly after birth, the neonate had tachypnea, cyanosis, intercostal indrawing, and one episode of low oxygen saturation. An air bronchogram over the retrocardiac region was noted on chest radiography and pneumonia was suspected. The patient was admitted to the newborn nursery and treated as sepsis/congenital pneumonia with intravenous gentamicin and ampicillin following sepsis work-up. She remained hemodynamically stable. Blood white cell count was raised at 14.6 × 10^9^/L while C-reactive protein plateaued at 9.85 mg/L. Lumbar puncture showed a protein level of 1.4 g/L and glucose level of 3.4 mmol/L. Cerebrospinal fluid (CSF) culture was negative.* Streptococcus agalactiae* was identified from the blood culture and the left ear skin swab. The patient was treated with intravenous ampicillin and cefotaxime for 5 days followed by intravenous benzylpenicillin for another 5 days. GBS was sensitive to penicillin G but resistant to clindamycin and erythromycin. Nonpneumococcal penicillin-sensitive streptococci could be considered susceptible to ampicillin, amoxicillin, cefazolin, cefuroxime, ceftriaxone, and carbapenems. Chest X-ray was repeated upon completion of antibiotic therapy and showed no abnormality. She was successfully breastfed and mother did not have mastitis. The infant was discharged at 14 days of age in good condition following counselling of the mother regarding the possibility of late onset GBS disease.

At 36 days of age, the patient presented to the emergency department with fever, irritability, poor feeding, and occasional regurgitation. Physical examination revealed an irritable child with a temperature of 39.1°C. Vital signs were stable. The fontanelle was full. Neurological examination was unremarkable. Intravenous penicillin with cefotaxime was given and the patient was immediately admitted to the pediatric intensive care unit. Investigations showed a white cell count of 14.2 × 10^9^/L and C-reactive protein of 102 mg/dL (which plateaued to 268 mg/dL). CSF showed a white cell count of 1420 × 10^9^/L with 89% polymorphs, protein of 3.8 g/L, and glucose less than 0.2 mmol/L. Blood glucose was 3.9 mmol/L. Gram stain of the CSF showed Gram-positive cocci. GBS, which was sensitive to penicillin but resistant to clindamycin and erythromycin, was positive in the CSF culture. Blood culture yielded no pathogen. The patient was treated with a 3-week course of intravenous antibiotics (penicillin and cefotaxime) and subsequently discharged after repeated sterile CSF along with stable vital signs. No neurological sequelae were detected. The infant remained well on follow-up 3 months later.

## 3. Discussion

Early onset GBS infection is defined as GBS infection during the first 6 days of life [1–3, 5, 6, 9]. The infection can be acquired through rupture of amniotic membranes (especially if prolonged) or during passage through the birth canal. The majority of neonatal GBS infections present in the initial 24 hours of birth with a percentage of 63.9% in one study [13]. Previous studies showed that the incidence rate was 0.58 cases among 1000 live births in Hong Kong [8]. This is comparable to the United States incidence of around 0.5 cases per 1000 live births in a study undertaken by the Centers for Disease Control and Prevention (CDC) in 2000 [1–3]. This figure has been on the decline since the 1990s when the incidence rate was at around 1.8 per 1000 live births in the US. The decline in early onset GBS infection can be attributed to the widespread use of intrapartum antibiotic prophylaxis and the universal screening of pregnant women for GBS colonization [1–3].

For early onset GBS, risk factors apart from maternal GBS colonization (around 20% of the general population) include premature birth, prolonged rupture of membranes, intrapartum maternal fever, and twin pregnancy [1–3, 8, 13]. A history of GBS disease in siblings of previous deliveries would also increase the probability of GBS disease [1]. The rates of neonatal septicemia for infants born to women diagnosed with vaginal GBS colonization are significantly elevated at 10 per 10,000 live births. Previous studies suggested the risk could be as high as 25 times compared to women who do not have vaginal GBS colonization [12, 13]. Vertical transmission of GBS usually occurs after rupture of the amniotic membranes or after onset of labor. In the present case, the source of GBS in the early onset GBS disease might be related to maternal vaginal colonization with GBS which was missed in the antenatal GBS screen. Alternatively, the source of the GBS might be from the maternal gastrointestinal colonization.

Late onset GBS disease is defined as infection with onset from 7 days to 89 days after birth [10, 14, 15]. If the onset occurs from 89 to 180 days after birth, it is known as late, late onset GBS (also called very late onset GBS or GBS beyond early infancy). The incidence of late onset GBS disease in the US was around 0.35 per 1000 live births in 1990 but declined to 0.27 per 1000 live births in 2013 [16].

Late onset GBS disease most commonly presents with septicemia, meningitis, and other focal infections such as cellulitis, lymphadenitis, and bone/joint infection [10, 14, 15]. Late and early onset GBS diseases share similar risk factors, but some crucial differences have been observed. The GBS colonization of maternal birth canal is not a crucial factor in late onset GBS disease as it is in early onset GBS disease although horizontal transmission from mother to infant is still possible after birth. As such, the use of maternal intrapartum chemoprophylaxis has been shown to be ineffective in the prevention of late onset GBS disease [11]. Rather, the most important maternal risk factor for late onset infections is premature birth of less than 37 weeks [10]. This could be explained by the reduced time for maternal IgG specifically for GBS to cross the placenta to the infant [17]. The greater the prematurity, the higher the likelihood for the infant to have later onset GBS infection. Additionally, the medical equipment used in supporting a preterm neonate could contribute as another source of infection [11]. Exposures to colonized parents and siblings are important risk factors for late onset GBS disease.

While late onset GBS disease is much less common compared to early onset GBS disease, its presentation and complications particularly neurological sequelae can be similarly devastating [2, 3, 10, 12]. In particular, worrisome is that the occurrence of late onset GBS disease does not appear to be as significantly reduced as in early onset GBS disease in the past decade [2, 3, 10]. It is therefore of prime importance to prevent the occurrence of late onset GBS disease after an early onset GBS disease. The use of intrapartum antibiotics for selective high-risk patients, while being sufficient for the prevention of early onset GBS disease, has been found to be insufficient for prevention of late onset GBS disease. In contrast, prematurity, the most critical factor for late onset GBS, will help to reduce the incidence of late onset GBS disease if it could be prevented [15]. Possible sources of infections must also be removed. Of particular interest are nosocomial cross-infections due to prolonged hospital stay, breastfeeding in the event of maternal mastitis, colonized household contacts, and poor hand hygiene of the caregivers [11]. Since oligosaccharides in breast milk can inhibit the growth of GBS, breast feeding in the absence of maternal mastitis should be strongly encouraged.

In the present case, the patient had both early and late onset GBS disease. The probability of reoccurrence after the first episode of GBS infection has been estimated to be between 1 and 6% depending on the specific localities. The mechanisms of reoccurrence are variable and not currently fully understood. Some authors have suggested the etiology to be due to suboptimal dosage of antibiotics used during the first infective episode leading to failure to eradicate the initial source of infection. When antibiotics are subsequently discontinued, the infection reemerges and thus becomes a reoccurrence. This would be especially significant in infants who are unable to produce the necessary antibodies and have a weaker immune response [14, 17]. This vulnerability would be exacerbated by the prematurity of the infant [14].

Other authors have suggested that the reoccurrence is due to a second source of infection. This is worth considering as many infants with early onset GBS disease would be exposed to many other sources and types of infection due to prolonged stay in the hospital [2, 3]. Alternative sources of infection include maternal mastitis in the case of breast feeding or simply poor hand hygiene among healthcare workers resulting in the spread of GBS in the ward [15].

In the management of this infant, cefotaxime and ampicillin were given before switching over to penicillin G. Penicillin G is generally recommended for the treatment of GBS infection as well as for use in pregnant women for intrapartum eradication of GBS colonization of the birth canal. Both erythromycin and clindamycin can be used in the case of penicillin resistance or hypersensitivity. This recommendation is supported by multiple guidelines and studies which have found GBS to be uniformly susceptible to penicillin and ampicillin. In contrast to penicillin, around 20 to 30% of GBS has been found to be resistant to both erythromycin and clindamycin [18]. Therefore, erythromycin and clindamycin should only be considered for GBS when both penicillin and ampicillin are unsuitable for use, for example, due to hypersensitivity [18, 19].

Early GBS disease may be prevented by intrapartum antibiotic, and affected infants can be treated by penicillin or ampicillin [1, 10]. Early detection of GBS is therefore essential. Given the rapid onset and progression within hours of birth and lack of an effective solution for preventing late onset GBS, administration of an effective vaccine in the third trimester of pregnancy could provide a sensible and cost-effective solution in all settings. The vaccine will protect neonates from GBS disease through transplacental transfer of antibodies to the fetus in utero. Protein conjugate vaccines and protein-based GBS vaccines are currently in development [17, 20]. Heath discussed a number of promising candidate vaccines [20]. In particular, phase I and II trials of a trivalent GBS vaccine have been conducted in >600 nonpregnant and >500 pregnant women in four countries to assess the optimal dose, need for adjuvant, immunogenicity in pregnant women, placental transfer, and persistence in babies. The trivalent vaccine appeared to be well tolerated and immunogenic.
